# MicroRNA-22 increases senescence and activates cardiac fibroblasts in the aging heart

**DOI:** 10.1007/s11357-012-9407-9

**Published:** 2012-04-27

**Authors:** Virginija Jazbutyte, Jan Fiedler, Susanne Kneitz, Paolo Galuppo, Annette Just, Angelika Holzmann, Johann Bauersachs, Thomas Thum

**Affiliations:** 1 Institute of Molecular and Translational Therapeutic Strategies (IMTTS), IFB-Tx, Hannover Medical School, Carl-Neuberg-Str. 1, 30625 Hannover, Germany; 2 Microarray Core Facility, Interdisciplinary Centre of Clinical Research, University of Würzburg, Würzburg, Germany; 3 Department of Cardiology and Angiology, Hannover Medical School, Hannover, Germany; 4 Centre for Clinical and Basic Research, IRCCS San Raffaele, Rome, Italy

**Keywords:** Cardiac aging, microRNAs, miR-22, Mimecan, Osteoglycin, Cardiac fibrosis

## Abstract

MicroRNAs (miRs) are small non- coding RNA molecules controlling a plethora of biological processes such as development, cellular survival and senescence. We here determined miRs differentially regulated during cardiac postnatal development and aging. Cardiac function, morphology and miR expression profiles were determined in neonatal, 4 weeks, 6 months and 19 months old normotensive male healthy C57/Bl6N mice. MiR-22 was most prominently upregulated during cardiac aging. Cardiac expression of its bioinformatically predicted target mimecan (osteoglycin, OGN) was gradually decreased with advanced age. Luciferase reporter assays validated mimecan as a *bona fide* miR-22 target. Both, miR-22 and its target mimecan were co- expressed in cardiac fibroblasts and smooth muscle cells. Functionally, miR-22 overexpression induced cellular senescence and promoted migratory activity of cardiac fibroblasts. Small interference RNA-mediated silencing of mimecan in cardiac fibroblasts mimicked the miR-22-mediated effects. Rescue experiments revealed that the effects of miR-22 on cardiac fibroblasts were only partially mediated by mimecan. In conclusion, miR-22 upregulation in the aging heart contributed at least partly to accelerated cardiac fibroblast senescence and increased migratory activity. Our results suggest an involvement of miR-22 in age-associated cardiac changes, such as cardiac fibrosis.

**Electronic supplementary material:**

The online version of this article (doi:10.1007/s11357-012-9407-9) contains supplementary material, which is available to authorized users.

## Introduction

Physiological aging and senescence are associated to progressive decline in most physiological functions, reduced response to stress, loss of viability and increased susceptibility to various diseases (Lakatta and Sollott 2002; Selvamurthy et al. 1999). Cardiac aging is associated with reduced cardiac functional reserve, left ventricular hypertrophy, mild fibrosis and reduced endogenous cardiac protection (Dai and Rabinovitch 2009; Jahangir et al. 2007). The incidence of cardiovascular disease increases dramatically with advanced age, therefore elucidation of the molecular mechanisms contributing to cardiac aging in healthy heart would help to identify early pathophysiological changes in the heart. Early diagnostics of cardiovascular disease might help to improve currently available therapies and develop new more efficient treatment strategies. The proposed mechanisms which are responsible for cardiac aging involve genetic and environmental factors, such as a decrease in telomerase activity and shortening of the DNA of the telomeres (Fuster and Andres 2006; Kajstura et al. 2006), increased oxidative stress (Terman and Brunk 2006), loss of mitochondrial function (Dai and Rabinovitch 2009) and impaired autophagy (Terman et al. 2007). In the last decade epigenetic regulators, such as small non- coding RNAs, called.g. microRNAs (miRs), were shown to be involved in cellular aging processes (Li et al. 2009; Williams et al. 2007). MiRs are endogenous 22-24 nt long RNA molecules which suppress target protein expression either by promoting messenger RNA (mRNA) degradation or by translational repression (Bauersachs and Thum 2011; Brodersen and Voinnet 2009). MiRs are highly conserved and account 1-5 % of worm, plant and vertebrate genomes (Blakaj and Lin 2008; Laporte et al. 2007). It is hypothesized that up to 90 % of human genes are regulated by microRNAs (Perron and Provost 2008). According to “microRNA.org” database (http://www.microrna.org/microrna/releaseNotes.do#GeneralInformation; August 2010 release), there are about 1100 human miRs with known sequences which are involved in nearly every developmental and cell function process, such as organogenesis and cell cycle, cell survival, apoptosis, migration and differentiation (Crespi and Frugier 2008; Fazi and Nervi 2008; Liu 2009; Thum et al. 2008a; Zhao and Srivastava 2007). MiRs are involved not only in physiological processes but are also major players in the onset and development of various diseases, such as cancer, immunological pathologies, neurodegenerative diseases and cardiovascular pathologies (Coolen and Bally-Cuif 2009; da Costa Martins et al. 2010; Dai and Ahmed 2011; Ferracin et al. 2010; Thum et al. 2008b). MiRs substantially contribute to the development of cardiac pathologies and their ability to influence gene networks suggest miRs as potential therapeutic targets and/ or diagnostic markers (Fichtlscherer et al. 2010; Thum et al. 2008b; Wang et al. 2010).

Here, we aimed to identify age- associated changes in miR expression profile in the murine heart and analyze prominent postnatally expressed and also aging-associated miRs. Additionally, we aimed to dissect molecular mechanisms and pathways that are regulated by aging- associated miRs in cardiac cells, especially cardiac fibroblasts.

## Results

### Morphometric and functional analysis of postnatal murine hearts during aging

Cardiac function and morphometric parameters of 4 weeks, 6 months and 19 months old animals were estimated by non- invasive echocardiography. Cardiac parameters, such as end- systolic area (ESA), and- diastolic area (EDA), interventricular septum thickness (IVS) and posterior wall thickness (PW) were significantly increased in aged animals compared to the parameters of 4 weeks old (control) animals (Table 1) Functional cardiac parameters, such as fractional shortening (FS) and heart rate (HR) remained unchanged between the groups (data not shown). Due to very small size of the neonatal mice (mean body weight 1.26 ± 0.013 g and mean heart weight 5.59 ± 0.3 mg), we here were not able to perform non-invasive echocardiography measurements. Morphometric measurements showed that absolute and relative heart weight gradually increased with advanced age (Table 2). Thus, echocardiography and morphometric measurements independently demonstrated age- dependent development of physiological hypertrophy in healthy male mouse hearts.

**Table 1:** Cardiac parameters measured by non- invasive echocardiography

Groups	Systole, AP ESA [mm2]	Diastole, AP EDA [mm2]	IVS [cm]	PW [cm]
4 weeks	0.038 ± 0.003	0.077 ± 0.005	0.119 ± 0.006	0.061 ± 0.007
6 months	0.059 ± 0.004	0.110 ± 0.004	0.150 ± 0.007	0.089 ± 0.005
19 months	0.056 ± 0.006	0.106 ± 0.006	0.154 ± 0.006	0.093 ± 0.003

Statistically significant parameters versus 4 weeks old animals (control group), p< 0.05

**Table 2:** Absolute and relative heart weight

Groups	Abs. HW [mg]	rel. HW [mg/ mm tibia]
Neonatal	5.59 ± 0.3	
4 weeks	80.03 ± 1.6	5.15 ± 0.1
6 months	137.54 ± 8.13	7.67 ± 0.51
19months	127.06 ± 3.63	7.13 ± 0.19

Statistically significant parameters versus neonatal animals (abs. HW) and 4 weeks old animals (rel. HW), p< 0.05

**Fig. 1:**
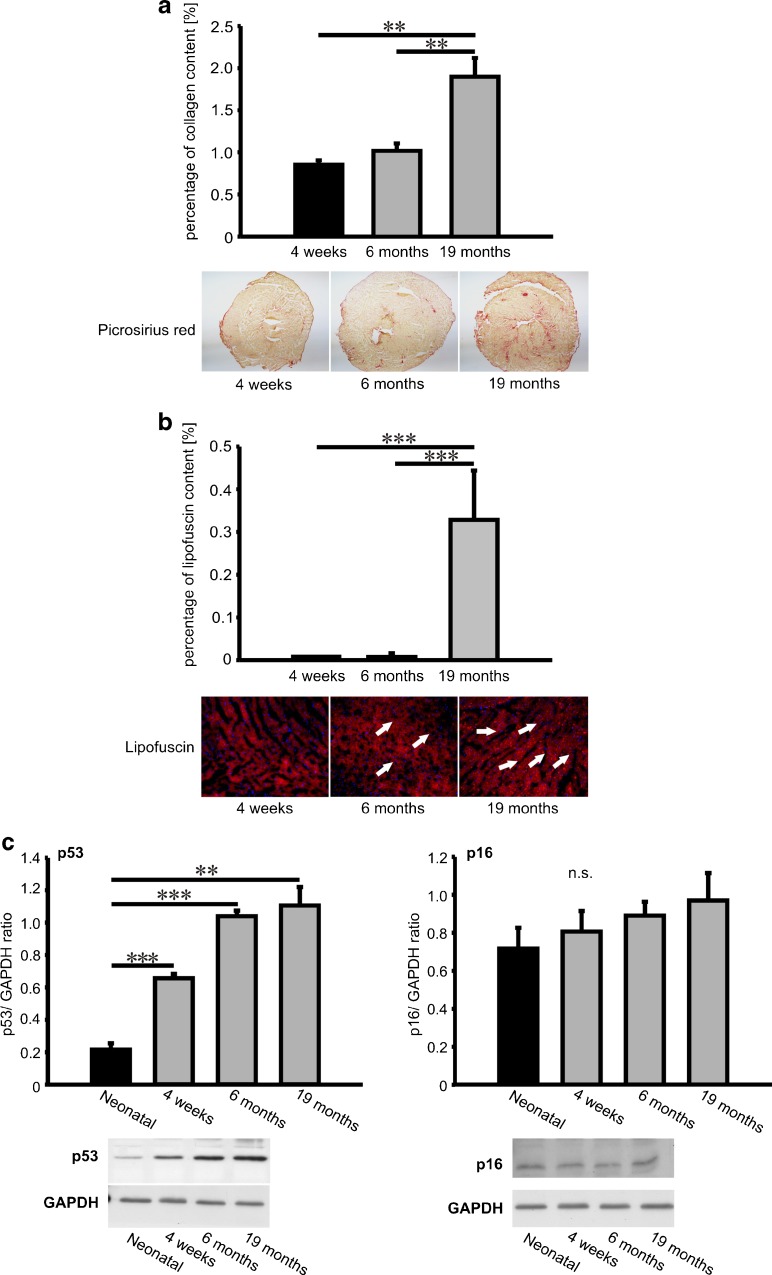
Cardiac fibrosis and aging (lipofuscin deposition, p53 and p16 expression). Cardiac fibrosis was visualized by picrosirius red (PSR) staining where collagen fibers are red whereas myocardium is stained in yellow. The degree of cardiac fibrosis is expressed as percentage of collagen signal versus total signal intensity (chart in Fig. 1a ). Representative PSR stainings are shown in the middle panel. Deposition of autofluorescent lipofuscin particles was observed in 4 % PFA fixated cryosections using fluorescence filter at 594 nm wavelength range (lower panel). Quantification data of lipofuscin signal intensity in cardiac sections are expressed as percentage of lipofuscin signal versus total signal intensity (chart, Fig. 1b). **c** Protein expression of senescence-associated p53 and p16 in cardiac tissue. Data in the chart are expressed as mean ± SEM (n = 4 tissues/sections/ group); **, p < 0.01; ***, p < 0.001

Collagen content measurements in 4 weeks old, 6 months and 19 months old mouse hearts revealed marginal collagen accumulation in 4 weeks and 6 months old mouse hearts and significantly increased cardiac fibrosis in 19 months old animals (3.8 % vs. 1.3 % in 4 weeks old hearts, p < 0.01) as depicted in Fig. 1a. Lipofuscin accumulation in the myocardium served as an independent parameter for cardiac senescence. Lipofuscin deposition started at the age of six months and reached its maximum in 19 months old hearts (Fig. 1, panel b). Senescence associated p53 and p16 levels were also increased during cardiac aging (Fig. 1, panel c).

### MicroRNA profiling in neonatal and postnatal murine hearts

**Fig. 2:**
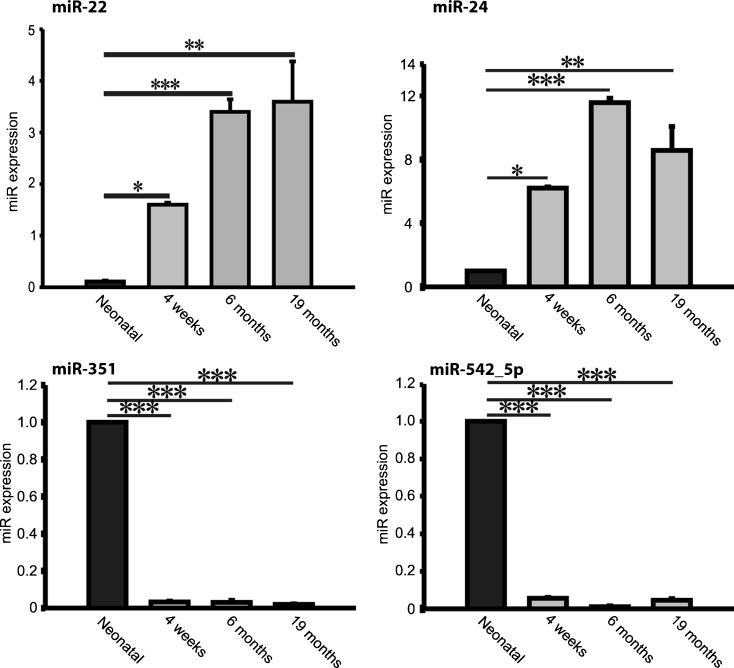
Postnatally differentially regulated microRNAs. Differentially expressed microRNAs in hearts from neonatal, 4 months, 6 months and 19 months old male Bl6 mice. MiR-22 and miR-24 were strongly upregulated whereas miR-351 and miR-542-5p were significantly downregulated in postnatal compared to neonatal hearts. Data are expressed as mean ± SEM (n = 8/ group). *, p < 0.05; **, p < 0.01; ***, p < 0.001

**Fig. 3:**
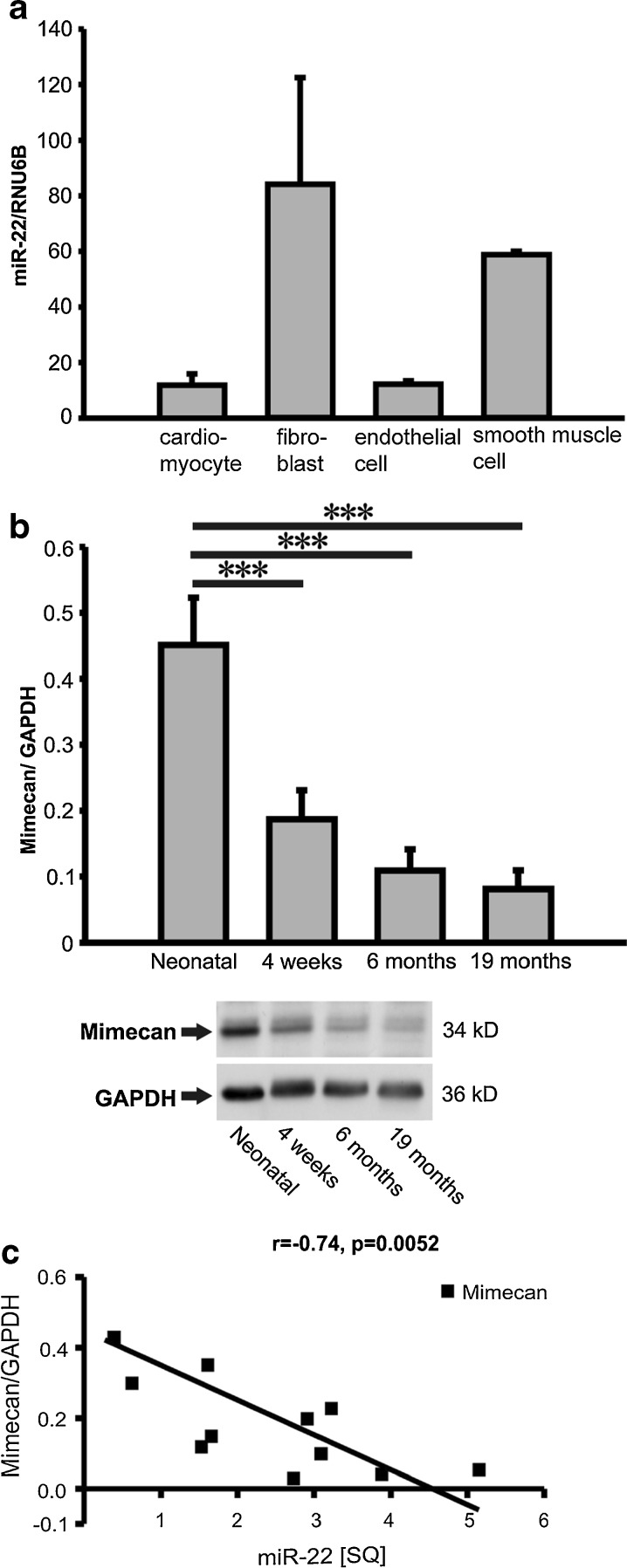
MiR-22 and mimecan expression in the aging heart. **a** MiR-22 expression in rat cardiac myocytes, human cardiac fibroblasts, umbilical cord endothelial cells and smooth muscle cells showed miR-22 enrichment in cardiac fibroblasts and smooth muscle cells (n = 3- 4/ group). **b** Protein expression levels of the miR-22 predicted target mimecan in hearts from aging mice. **c** Negative correlation between miR-22 and mimecan in the mouse hearts depicted by regression analysis with a Pearson’s correlation coefficient of r = -0.74 (p = 0.0052). Data are expressed as mean SQ ± SEM. *, p < 0.05; **, p < 0.01; ***, p < 0.001

MicroRNA expression profiles in hearts of neonatal, 4 weeks old, 6 months and 19 months old mice were assessed using a miR array approach (data not shown). We then used a set of neonatal, 4 weeks old, 6 months and 19 months old mice for further validation analyses. MiR-22 and miR-24 were highly upregulated, whereas miR-351 and miR-542-5p were significantly downregulated in post-natal hearts compared to neonatal controls (Fig. 2). For further analysis, we focused on miR-22 whose expression positively correlated with advanced age in the various studied groups (Fig. 2). Various cardiac cell types, such as neonatal rat cardiomyocytes, human cardiac fibroblasts, smooth muscle cells and endothelial cells were analyzed for miR-22 expression levels. MiR-22 was enriched in cardiac fibroblasts and smooth muscle cells, whereas lower miR-22 levels were observed in cardiomyocytes and endothelial cells (Fig. 3a).

### Mimecan is a direct target of miR-22 in the mouse heart

To identify miR-22 targets, we first performed computational miR target analysis. Three microRNA target prediction programs (PicTar, TargetScan and microRNA.org) revealed mimecan (osteoglycin) as a potential miR-22 target gene. We performed mimecan protein expression analysis in the mouse hearts of different age groups and observed mimecan to be highly expressed in the neonatal heart but gradually decreased during cardiac aging (Fig. 3b). Indeed, expression levels of miR-22 and mimecan showed a highly significant inverse correlation (r = -0.76, p < 0.0052) suggesting that mimecan may be a target for miR-22 in the heart (Fig. 3c).

**Fig. 4:**
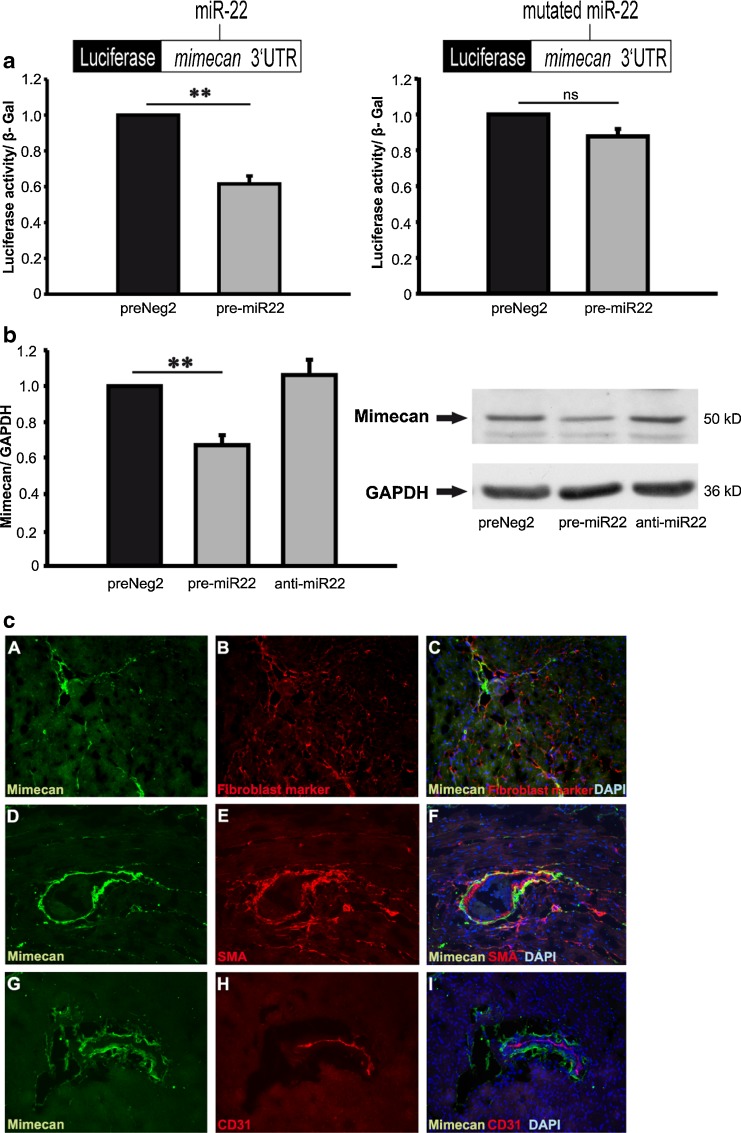
Mimecan is regulated by miR-22 and localized in the mouse heart. **a** Luciferase reporter vector carrying either wild- type (left panel) or mutant human mimecan 3’- UTR (right panel) was co-transfected with control miR (preNeg2) or miR-22 precursor (pre-miR22). Luciferase activity results were normalized versus beta galactosidase activity in the same cells. **b** MiR-22 overexpression resulted in significant mimecan protein downregulation in cardiac fibroblasts, whereas inhibition of endogenous miRNA by sequence- specific antagonist did not influence mimecan expression. Representative mimecan western blots are shown in **b**, right panel. **c** Immunofluorescent staining of mimecan protein expression (green) in the murine heart. Mimecan was partially co- localized with cardiac fibroblasts (red) (panels **a-c**) and smooth muscle cells (red) (smooth muscle actin (SMA) staining) (panels **d-f**) but not with endothelial cells (red) (marker CD31) (**g-i**). Cell nuclei were stained with DAPI. The pictures were taken at 200x magnification. Data are expressed as mean ± SEM (n = 3-5/ group). **, p < 0.01

Direct mimecan regulation by miR-22 was analyzed using a luciferase reporter gene approach. Two luciferase reporter gene constructs carrying either wild-type (wt) 3’- UTR of murine mimecan gene (OGN) or mutant (mut) 3’-UTR carrying three nucleotide changes within the miR-22 binding site of OGN 3’- UTR, were generated to analyze miR-22 binding to wt and mut OGN 3’- UTRs (Fig. 4a). HEK293 cells were co- transfected with either wt or mut OGN 3’-UTR together with miR-22 precursors (pre-miR-22) or control precursor miRs (preNeg2). Luciferase activity was assessed 24 hours post-transfection and normalized against beta- galactosidase activity. The results showed that overexpressed miR-22 efficiently downregulated OGN wt 3’-UTR luciferase reporter activity reaching approximately 40 % compared to control miRs (Fig. 4a, left panel). In contrast, mutation of three nucleotides in miR-22 binding site in the OGN 3’-UTR (OGN mut) abrogated miR-22 precursor- dependent reduction of luciferase reporter activity (Fig. 4a, right panel). We further studied mimecan protein expression changes after miR-22 modulation in cardiac fibroblasts which were transfected with control miR (preNeg2), miR-22 precursor (pre-miR-22) or miR-22 antagonist (anti-miR-22). Overexpression of miR-22 in cardiac fibroblasts resulted in significant decrease in mimecan expression whereas inhibition of endogenous miR-22 in fibroblasts did not affect mimecan expression (Fig. 4b). Next, we analyzed mimecan localization pattern in the mouse heart and observed mimecan to partially co-localize with cardiac fibroblasts and within perivascular areas (Fig. 4c, inlays a-c) as well as with smooth muscle cells, which were located in larger cardiac vessels (Fig. 4c,inlays d-f). In contrast, there was no co-localization between mimecan and endothelial cells or cardiomyocytes (Fig. 4c, inlays g-i and data not shown).

In conclusion, we identified cardiac miR-22/mimecan co-expression and validated mimecan to be a direct target of miR-22 in cardiac fibroblasts.

### MiR- 22 and mimecan regulate cellular senescence and chemotaxis

**Fig. 5:**
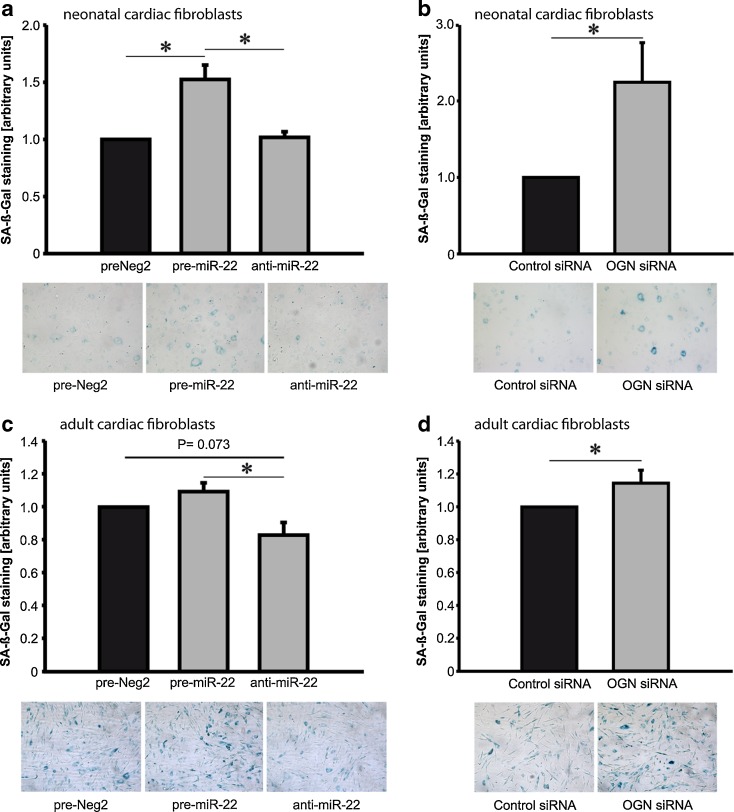
Effects of miR-22 on cellular senescence. Neonatal rat and adult human cardiac fibroblasts were transfected with miR-22 precursor (pre-miR-22), miR-22 antagonist (antimiR-22) or control miR (preNeg2) (**a, c**). To determine enzymatic activity of senescence-associated beta galactosidase (SA-β- Gal), cells were fixed and incubated with β- Gal substrate. Blue color intensity was analyzed and data was expressed as ratio between treatment and control group. MiR-22 overexpression significantly induced premature senescence in neonatal rat cardiac fibroblasts (**a**). Mimecan (osteoglycin, OGN) silencing using sequence- specific siRNA induced cellular senescence in neonatal fibroblasts mimicking the effects of miR-22 overexpression (**b**). Adult cardiac fibroblasts expressed high endogenous levels of miR-22, thus overexpression of this microRNA resulted in marginal increase in cellular senescence, whereas miR-22 inhibition reduced it (**c**). Mimecan (OGN) silencing increased cellular senescence in adult cardiac fibroblasts (**d**). Data are expressed as mean ± SEM (n = 4-5/ group). *, p < 0.05

To determine which cellular functions might be affected by miR-22, we studied potential changes in cellular senescence, chemotaxis, proliferation, apoptosis and cell migration of cardiac fibroblasts. To assess cellular senescence, we analyzed senescence- associated beta galactosidase (SA-β- Gal) activity in neonatal rat cardiac fibroblasts and human cardiac fibroblasts (HCFs). MiR-22 overexpression in neonatal cardiac fibroblasts resulted in a significant increase in senescent cells whereas inhibition of this miR by a sequence-specific antagonist did not affect cellular aging (Fig. 5a). MiR-22-mediated increase in cellular senescence could be mimicked by mimecan silencing where mimecan-deficient fibroblasts were more prone to premature senescence compared to control-siRNA transfected fibroblasts (Fig. 5b). In adult cardiac fibroblasts, which express high levels of endogenous miR-22, ectopic overexpression of miR-22 only slightly affected cellular aging. However, suppression of endogenous miR-22 using miR-22 specific antagonist significantly reduced SA-β- Gal positive cell number indicating that miR-22 directly contributes to cellular senescence (Fig. 5c). Mimecan silencing similarly upregulated cellular senescence in cardiac fibroblasts thus mimicking the miR-22 effects (Fig. 5d). There was a trend for increased expression of senescence-associated p16 expression in cardiac fibroblasts overexpressing miR-22 or after mimecan silencing (Supplementary Figure 1a and b). We next constructed experiments to test the direct role of mimecan for the miR-22-mediated effects in cardiac fibroblast. The efficiency of mimecan knockdown / viral overexpression was assessed by western blotting as indicated in Supplementary Figure 2. First, miR-22 depletion efficiently reduced cellular senescence but additional silencing of mimecan had no rescuing effects (Supplementary Figure 3a). Second, in adult fibroblasts overexpressing miR-22, the co-transfection of a miR-22-resistant mimecan construct only partly rescued the effects on cellular senescence (Supplementary Figure 3b). These results point to miR-22 as an important regulator of cellular senescence and that only parts of its effects are directly mediated via mimecan. This is likely as a single miR may have hundreds of different targets and thus further targets mediating miR-22 effects in cardiac fibroblasts remains to be determined.

**Fig. 6:**
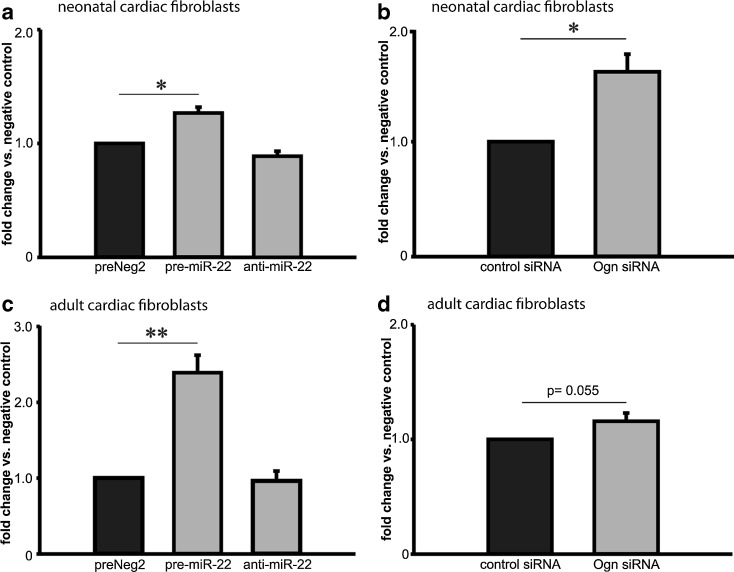
Chemotactic migratory capacity of cardiac fibroblasts is mediated by miR-22**.** Neonatal rat and adult human cardiac fibroblasts were transfected with miR-22 precursor, antagonist and control miR (preNeg 2). Chemotactic migration assay was performed using modified Boyden chamber approach where cells migrated through semi- permeable membrane towards 20 % FCS. Migrated cells were stained with DAPI and counted. The mean number of migrated neonatal fibroblasts transfected with control miRNA was set to 1 and the ratios between pre-miR-22 and anti-miR-22 versus group and control are depicted in panel **a**. MiR-22- induced chemotactic activity of fibroblasts could be mimicked by mimecan (Ogn) knockdown in neonatal fibroblasts (**b**). Similarly, ectopic expression of miR-22 in adult fibroblasts also resulted in significant increase in chemotaxis (**c**) and these effects could be reproduced by mimecan (Ogn) knockdown (panel **d**, p = 0.055). Data are expressed as mean ± SEM (n = 4-5/ group). *, p < 0.05

Fibroblast chemotactic activity was assessed using a modified Boyden chamber assay. We transfected neonatal and adult cardiac fibroblasts with control miR, miR-22 precursor or miR-22 antagonist, and let the cells migrate towards 20 % FCS. Both neonatal and adult cardiac fibroblasts overexpressing miR-22 showed up to 150 % increased chemotactic migratory activity compared to control cells (Fig. 6a-d). To test the influence of mimecan in the miR-22-mediated effects on fibroblast chemotactic activity we first silenced miR-22 via treatment with anti-miR-22. This resulted in a trend for lower migratory activity and additional silencing of mimecan via siRNA had only little effect (Supplementary Figure 4a). Next, we overexpressed miR-22 leading to increased chemotactic activity. Co-transfection with a miR-22-resistant form of mimecan did not reverse the pro-chemotactic effects of miR-22 (Supplementary Figure 4b). Both experiments show that miR-22 effects on chemotactic activity are independent of mimecan.

We also tested the effects of miR-22 overexpression in smooth muscle cells, where we observed a high senescence- associated β-Gal activity whereas miR-22 inhibition reduced cellular senescence in this cell type (Supplementary Figure 5a). A similar increase in chemotactic migration was observed in smooth muscle cells overexpressing miR-22 (Supplementary Figure 5b). Another cell migration assay, the scratch wound assay, was performed to test, whether miR-22 influences cell migration independent from chemotactic stimuli. To prevent cell proliferation, mitomycin C was added to the cell culture medium. Here, we did not find differences in cell migration in neonatal or adult fibroblasts (data not shown). Likewise, cell proliferation and cell apoptosis/necrosis measurements indicated no significant functional changes in all treatment groups and both cell types (data not shown).

In conclusion, we here show miR-22 upregulation during cardiac aging and demonstrated that miR-22 selectively regulates cellular senescence and cellular chemotactic migration in cardiac fibroblasts which may contribute to miR-22 dependent cardiac fibrosis during aging.

## Discussion

In our study, we demonstrate for the first time that miR-22 contributes to cardiac aging by influencing cardiac fibroblasts. It was recently demonstrated that miRs, such as miR-19b, miR-20a and miR-106a were differentially regulated in old human cells, such as endothelial cells, CD8+ T- cells, renal epithelial cells and skin fibroblasts (Hackl et al. 2010). Van Almen and colleagues demonstrated that the members of miR-17-92 cluster, miR-18a, -19a and -19b, target extracellular proteins such as connective tissue growth factor (CTGF) and thrombospondin-1 (TSP-1) which were upregulated in old failing hearts (van Almen et al. 2011). Other miRs, such as miR-21 and the members of miR-29 family, were shown to be involved in extracellular matrix remodeling in the heart where miR-21 promoted fibrosis and miR-29 family members were negative regulators of cardiac fibrosis following myocardial infarction (Thum et al. 2008b; van Rooij et al. 2008).

The approach of miR screening in the heart revealed several miRs whose expression was tightly associated with the cardiac aging. MiR-22 expression was progressively increasing with advanced age suggesting a role in cardiac aging. We identified mimecan/ osteoglycin (OGN) as a novel target for miR-22 in the mouse heart. Mimecan is a secretory protein which belongs to small leucin- rich proteoglycan family and was first described together with TGF-β1 and TGF-β2 to be involved in ectopic bone formation (Bentz et al. 1989; Madisen et al. 1990). Later, mimecan was detected not only in the bone, but also in the vascular matrix (Shanahan et al. 1997), cornea (Dunlevy et al. 2000), skin (Tasheva et al. 2002), the auditory system (Williamson et al. 2008) and other organs. In the present study we show that mimecan is expressed in the mouse heart with highest expression levels in the neonatal hearts and its expression gradually decreases with advanced age due to increased miR-22 expression. Mimecan localization studies showed that mimecan was co-expressed with miR-22 in cardiac fibroblasts and smooth muscle cells but we could not detect significant mimecan expression in cardiomyocytes, as described recently (Petretto et al. 2008). The variations of mimecan localization in the cardiac cells might be species-specific.

We could induce premature senescence in neonatal rat cardiac fibroblasts by miR-22 overexpression. Our results are in agreement with recently published data where miR-22 was shown to be upregulated in human senescent fibroblasts and epithelial cells and downregulated in various cancer cell lines (Xu et al. 2011a). The authors also identified CDK6, SIRT1 and Sp1 as miR-22 targets and postulated that miR-22 act as tumor suppressor. We studied SIRT1 as putative protein target for miR-22 in the heart and determined endogenous SIRT1 levels in all four age groups but could not find significant differences during aging (data not shown). However, there might be additional functions of this miR in other cardiac cell types, such as cardiomyocytes. Indeed, miR-22 overexpression also results in cardiomyocyte hypertrophy (Jentzsch et al. 2011; Xu et al. 2011b), although this is not the topic of the present manuscript where we mainly focused on cardiac fibroblasts. Indeed our data showed that miR-22 overexpression increased cellular senescence which is in a good agreement with recently published data (Xu et al. 2011a). However, we only observed a trend for senescence-associated p16 in fibroblasts after mir-22 overexpression or mimecan silencing potentially suggesting longer time periods are needed to fully induce a senescence-associated phenotype (Supplementary Figure 1). In contrast to the described anti-proliferative capacity of miR-22 in hepatocellular carcinoma cells, we did not observed any changes in cell proliferation following miR-22 modulation in cardiac fibroblasts and smooth muscle cells suggesting cell-type specific effects (Zhang et al. 2010). We found that cardiac fibroblasts and smooth muscle cells overexpressing miR-22 demonstrated enhanced chemotactic migration. This data suggests that miR-22- dependent cell movements are rather cell surface receptor dependent than based on cell- cell interactions as we did not see migration differences as in a (chemotactic independent) scratch wound migration assay. Indeed, data about the role of chemotaxis in the aging heart are sparse. In contrast, there is much information about chemotaxis in the context of inflammation events associated with cardiovascular disease (Madorin et al. 2004). Mimecan depletion in neonatal and adult cardiac fibroblasts mimicked the effects of miR-22 overexpression showing accelerated cellular aging and increased chemotaxis. However, whereas parts of the effects of miR-22 on cellular senescence might be mediated via mimecan, its effects on fibroblast-mediated activity were mimecan-independent. Thus, further, as yet unidentified, targets of miR-22 are likely to be involved in its fibroblast-specific effects.

Taken together, we demonstrate that cardiac miR-22 and mimecan expression was tightly associated with cardiac aging. We show that both molecules affect fibroblast aging and are involved in fibroblast activation without affecting cell proliferation and apoptosis. Future *in vivo* and *in vitro* studies are required to assess further molecular mechanisms of cardiac aging which are affected by miR-22 and its downstream target mimecan. Whether miR-22 based treatment strategies would affect cardiac aging and/or cardiac fibrosis is a challenging questions that awaits future analysis.

## Experimental procedures

### Materials and chemicals

A detailed list of the materials and chemicals used in this study is presented in *Supplementary data.*

### Mice

This study was approved by the local animal care committee of the local government (Regierung Unterfranken; Germany). The studies were performed in accordance with the guidelines published by the US National Institutes if Health (NIH publication Nr. 85-23, revised 1996). Male C57/Bl6N mice (4 weeks old, 6 months old and 19 months old) were obtained from Charles River Wiga GmbH (Sulzfeld, Germany). Neonatal mice (1-2 days old) were obtained from the animal facility of the University Clinics in Würzburg, Germany. The 4 weeks old, 6 months old and 19 months old mice were kept for one week under standard day/ night cycle, the animals became standard chow and water *ad libitum*. Cardiac function and heart size was monitored by non-invasive echocardiography which was performed by a trained person. Morphometric data, such as cardiac weight, body weight, lung weight and tibia length were collected to analyze cardiac morphometric parameter changes during the aging. For molecular analysis and histological studies, the hearts were explanted, weighted and cut in three pieces which were stored in liquid nitrogen for RNA and protein extraction or frozen in TissueTec for histological analysis.

### Cell culture

Human cardiac fibroblasts (HCF) were cultivated in Fibroblast Basal medium supplemented with 5 % and human aortic smooth muscle cells (HASMC) were grown in SmbM2 medium supplemented with hFGF4, insulin and 10 % FCS. Neonatal rat cardiac fibroblasts and HEK293 cells were cultivated in DMEM medium containing 10 % FCS. For starvation experiments, the cells were cultivated in the respective medium without supplements, such as growth factors, and with 1/10^th^ of the initial FCS concentration for 24 h before begin of the experiments.

### *In vivo* studies

Cardiac function and heart size was monitored by non- invasive echocardiography. Briefly, animals were placed on heating pad, anesthesized by isoflurane and oxygen mixture and cardiac dimensions and function were assessed by pulse- wave Doppler echocardiography using Toshiba PowerVision6000 system and a 15 MHz transducer. Cardiac parameters, such systolic and diastolic area, interventricular septum and posterior wall thickness as well as functional parameters, such as heart rate and fractional shortening were measured to assess cardiac hypertrophy and function. The data were analyzed using NICE TOS- eBASE software (Toshiba Medical Systems, The Netherlands) in a blinded fashion.

### RNA preparation

Total RNA from the mouse heart was extracted using Trizol® reagent according to the manufacturer’s recommendations. Briefly, frozen tissue was homogenized in Trizol and total heart RNA was extracted as described in the protocol. RNA quality was assessed using 2100 Bioanalyzer from Agilent in combination with Agilent RNA 6000 Nano Kit. High quality total RNA was further process to enrich microRNAs. MicroRNA enrichment was performed using flashPAGE Fractionator Sytem (Ambion) according to manufacturer’s protocol. Enriched small- molecular weight RNA was further subjected to microRNA arrays. Total RNA from cells and cardiac tissue was extracted using Trizol® reagent according to standard protocol, the RNA quality and concentration was defined spectrophotometrically and the samples were kept at -80°C.

### MicroRNA arrays

MicroRNA expression profile in the neonatal, juvenile, adult and senescent mouse hearts was analyzed using microRNA arrays containing “mirVana™ miRNA probe set”. Three arrays per age group were hybridized with the RNA probes where each probe contained pooled RNA from three animals from the same group. MicroRNA samples were labeled using “mirVANA™ miRNA labeling kit” and hybridized with microRNA arrays according to the manufacturer’s recommendations. Densitometric analysis (gridding) was performed using free software “ScanAlyze” (Stanford University, USA), and statistical data analysis was performed by blinded person (statistics with “R”). The microRNAs which were at least 1.5- fold differentially regulated compared to microRNAs from neonatal (control) group, were selected for further analyses.

### Quantitative PCR (qPCR)

Differentially regulated microRNAs were validated by quantitative real-time PCR and Taqman assays. Briefly, first- strand DNA synthesis using microRNA specific primers was performed using iScript™ Select cDNA synthesis system. The microRNA signal was amplified and detected using iQ™ Supermix and microRNA- specific TaqMan® MicroRNA probe under standard conditions described by Bio-Rad. The microRNA expression results were normalized against house keeping gene, RNU6B, and the data was analyzed using ΔΔC(t) and starting quantity methods.

### Western blotting

Western blotting was performed as described otherwise. Briefly, heart tissue was homogenized in lysis buffer containing protease inhibitors and DTT, centrifuged and protein concentrations were measured using Bradford method. Total protein (40 μg) was loaded on polyacrylamide gel, separated under reducing conditions and transferred to PVDF membrane. After transfer, the membranes were blocked with 5 % non- fat milk solution and incubated overnight with primary antibodies against Mimecan, SIRT1 and GAPDH at concentration 1 μg/ ml. Next day, the membranes were washed with TBS/T and incubated with anti- goat and anti- mouse HRP- conjugated secondary antibodies for one hour at room temperature, washed with TBS/T, incubated with ECL reagent and visualized using X-ray film. The X-ray film was developed, scanned and the signal was quantified using “Scion Image” freeware (Scion Corporation). Protein of interest signal was normalized versus house keeping gene GAPDH.

### Picrosirius red staining

Collagen content in mouse heart cryosections was visualized by picrosirius red staining according to the standard protocols. Cryosections were fixed with 37 % formaldehyde and incubated in picrosirius red solution for 1 h. Subsequently, the sections were rinsed with diluted acetic acid and, washed with water and mounted with Entellan. The percentage of collagen content in the sections was determined densitometrically using Adobe Photoshop CS3 software.

### Lipofuscin visualization

Mouse heart cryosections were dried, fixed 10 minutes with 4 % paraformaldehyde (PFA) solution and mounted with Vectashield medium containing DAPI. Lipofuscin autofluorescence and DAPI was observed at 600 nm and 460 nm, respectively and photographed using Nikon Ti microscope. Lipofuscin signal quantification was performed using Adobe Photoshop CS3 software and expressed as percentage of red signal versus global pixel number.

### Immunohistochemistry

Cardiac tissue samples were frozen in Tissue-Tek® O.C.T. compound, cut in 5- 10 μm cryosections, placed on SuperFrost Plus slides and air- dried. The sections were fixed with 4 % PFA and permeabilized with 0.1 % Triton X-100. Subsequently, unspecific background was blocked with 5 % donkey serum and sections were incubated overnight with goat- anti- mimecan, rat- anti- fibroblast marker, rat- anti- CD31 diluted in 5 % donkey serum at concentration 3-5 μg/ml. Next day, the sections were washed with PBS and incubated with the fluorescence- conjugated secondary antibodies (donkey- anti- goat AlexaFluor 488, donkey- anti- rat AlexaFluor 594 and donkey anti- rabbit AlexaFluor 594) together with DAPI for nuclear staining. The sections were mounted using VectaShield® mounting medium, covered with cover slips and subjected to fluorescence microscopy. Fluorescence microscopy pictures were taken by Nikon Ti fluorescence microscope and analyzed using NIS-Elements BR 3.2 software (Nikon corp.).

### Cell transfection

HCFs, neonatal rat cardiac fibroblasts and HASMCs were transfected with miR-22 precursor or miR-22 antagonist together with the scrambled control (100 nM) as well as siRNA against human osteoglycin and control siRNA (3.3 nM of each duplex and end concentration of 10 nM) using Lipofectamine 2000 reagent. The procedure was performed as described by manufacturer. The cells were transfected for 72 h and subsequently subjected to further cellular assays.

### Adenoviral transduction

Human cardiac fibroblasts were infected with an adenovirus carrying the human OGN gene under control of CMV promoter (Ad-OGN). The viral construct was applied following 4 hour transfection with miR-22 precursors and/or antagonists and the cells were incubated in diluted adenovirus overnight. Working concentration of adenovirus was 250 MOI, which was determined during serial dilution experiments. The cells were transduced/transfected for 72 hours and subsequently subjected to various cellular assays. Anadenovirus carrying the GFP gene under control of CMV promoter served as a negative control for the Ad-OGN construct. The efficiency of osteoglycin (mimecan) overexpression in cardiac fibroblasts is demonstrated in Supplementary Figure 2.

### Luciferase reporter gene assay

Murine OGN 3´-UTR (fragment of 569 bp) containing a miR binding site for miR-22 was PCR amplified and cloned into *SpeI* and *HindIII* digested pMIR-REPORT vector. For mutagenesis of the miR-22 binding site, the QuikChange Multi Site-Directed Mutagenesis Kit was applied according to manufacturers´ instructions. The resulting construct was co-transfected with miRs and beta-galactosidase control plasmid into HEK293 reporter cells in 48-wells using Lipofectamine 2000, 20 ng plasmid DNA and 100 nM miR. Cells were incubated for 24 h before measuring luciferase and β-galactosidase activity with appropriate substrates.

### Senescence- associated beta galactosidase (SA-β- Gal) assay

SA-β- Gal activity at pH 6.0 was histochemically detected using Senescence-β- Galactosidase assay according to manufacturer’s instructions. Briefly, the cells were grown in 6- well plates, transfected and 72 h later, fixed with 1x Fixative solution and incubated overnight in staining solution containing X-gal. Next day, staining solution was discarded and cells coated with 70 % glycerol. Cells were photographed at 10× magnification using Nikon Ti microscope and blue signal intensity was quantified densitometrically. Briefly, the .bmp images were analyzed using Adobe Photoshop CS3 software where intensity of the blue color i.e. SA-b-Gal signal was assessed. The pictures from one experiment were analyzed using the same settings and differences between treatment groups and control group was expressed as a ratio versus control group which was set to one. Five areas of each well representing one experimental condition were photographed and quantified. Mean signal intensity of four and more independent experiments was analyzed statistically.

### Chemotaxis assay (modified Boyden chamber)

Prior to the assay, transfected cells were stained with DAPI directly in the cell culture plate, trypsinized, counted and 50000 cells were applied to the HTS Fluoro Block inserts with the 8 μm pore size which outer side of the membrane was coated with fibronectin. The cells migrated towards cell culture medium containing 20 % FCS for 24 hours or shorter, if indicated otherwise. The fluorescence microscopy pictures were taken by Nikon Ti fluorescence microscope, the nuclei in the pictures were counted by blinded person. Each treatment group was tested in triplets and mean numbers of migrated cells of three and more independent experiments were analyzed statistically.

### Scratch wound assay

The cells were cultivated in 6- well plates and scratch in cell monolayer was performed using 100 μl pipette tip. The cells were kept in the cell culture medium containing mitomycin C (5 μg/ ml) to prevent cell proliferation. Scratches were photographed at timepoint zero (0 h), after 6 hours and 24 hours. Scratch area was estimated using NIS Elements BR 3.2 software and the ratio between scratch area at timepoints 6 hours and 24 hours served as surrogate parameter for cell migration capacity. The influence of miR-22 and Ogn modulation on cell migration was assessed comparing scratch area of miR-22 and Ogn si- modulated cells at one timepoint versus respective negative controls which were set to 100 %. The results are presented as percent difference between control and treatment group.

### Cell proliferation assay

Cell proliferation capacity was determined using WST-1 reagent. Briefly, the cells were seeded in 96- well plates, transfected and grown for 72 hours. Before cell treatment, WST-1 reagent was diluted in the cell culture medium at final concentration 1:10 and immediately applied to the cells. The absorbance at time points zero minutes, 30 minutes and 1 hour was measured at 450 nm wave-length using a microplate reader Synergy HT (Biotek, Bad Friedrichshall, Germany). Cell proliferation rate was calculated as a ratio between 1 hour and 30 min. and timepoint zero and compared between different treatment groups.

### Apoptosis assay

Apoptotic cells were stained using Annexin-V- FLUOS Staining system according to manufacturer’s recommendations. Apoptotic cell detection was performed using flow cytometer FACSCalibur (BD Biosciences, Heidelberg, Germany) and quantitative analysis was performed using CellQuest™ software.

### Statistics

Statistical analysis was performed using SigmaStat 2.0 software (Systat GmbH) where, dependent on the group number, ANOVA or t-test were applied. The post-hoc analyses were used in combination with ANOVA:

A P value < 0.05 was considered as statistically significant.

## Electronic supplementary material

Below is the link to the electronic supplementary material.

ESM 1 (DOC 1439 kb)

## Abbreviations

miR-22: microRNA 22
Ogn: osteoglycin/ mimecan
