# Supplementary material for: MicroRNA-22 increases senescence and activates cardiac fibroblasts in the aging heart
Source: Age (Dordr). 2012 Apr 27;35(3):747–62. doi: 10.1007/s11357-012-9407-9 (PMC3636396; doi:10.1007/s11357-012-9407-9)
Supplement: Supplementary file 1 — (DOC 1439 kb) [file 11357_2012_9407_MOESM1_ESM.doc]

# Supplemental Information

# Supplementary Figure Legends

Supplementary Figure 1. Protein expression of p16 via miR-22 and mimecan modulation in human cardiac fibroblasts. p16 protein expression was analyzed in cardiac fibroblasts overexpressing miR-22 (pre-miR-22) or deficient in mimecan/ OGN (OGN siRNA) (A and B, respectively). Data are expressed as mean ± SEM (n= 4/ group).


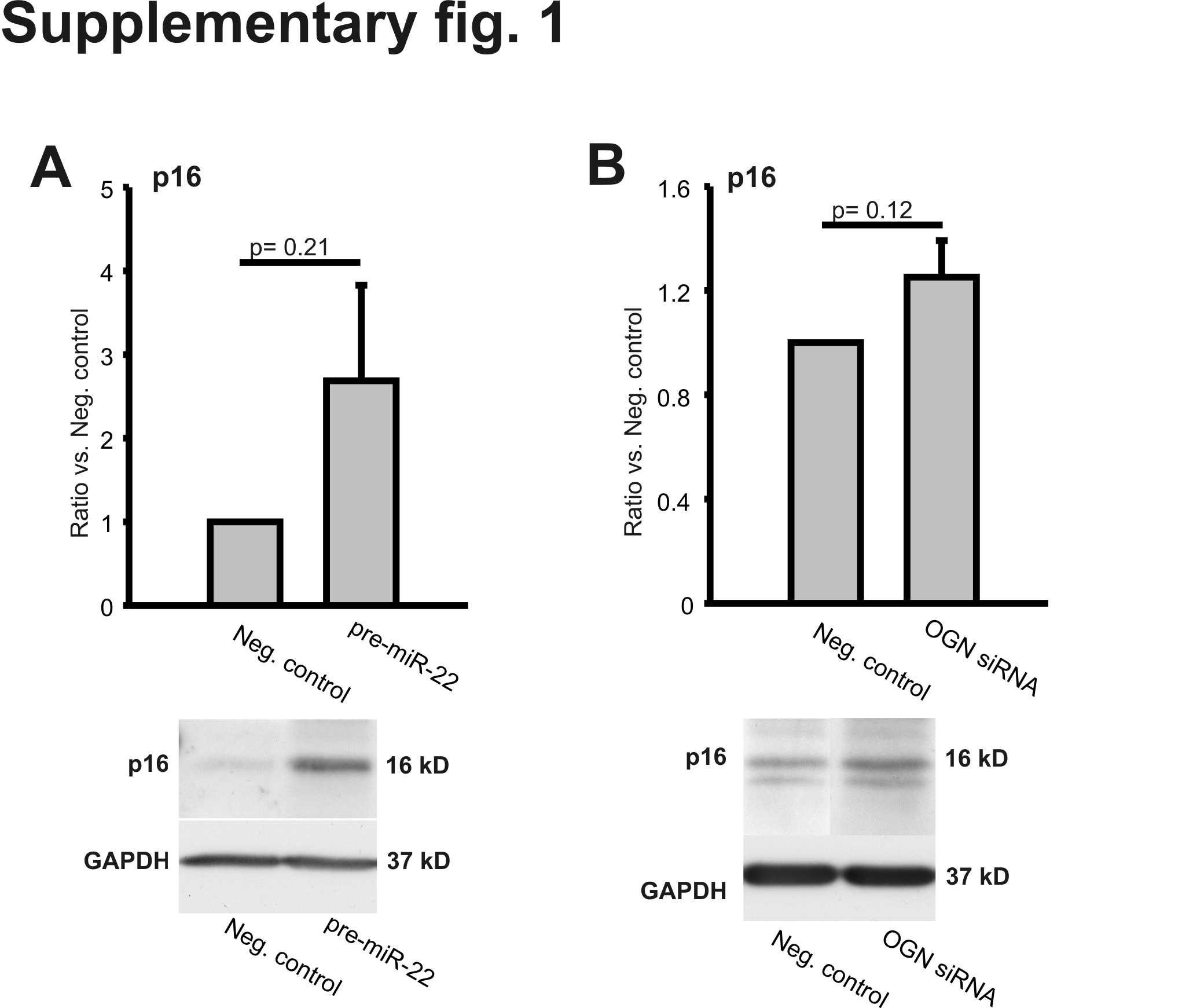


Supplementary Figure 2. Western blots showing mimecan knockdown and overexpression efficacy in cardiac fibroblasts. In mimecan (OGN) knockdown experiments, human fibroblasts were transfected with OGN siRNA(10 nM) for 72 h and protein expression was analysed by western blotting using specific antibody (A). In the OGN overexpression approach, fibroblasts were infected with adenovirus coding for human OGN cDNA at a concentration of 250 MOI for 72h. Subsequently, protein overexpression was assessed by western blot using specific antibody against human OGN (B).


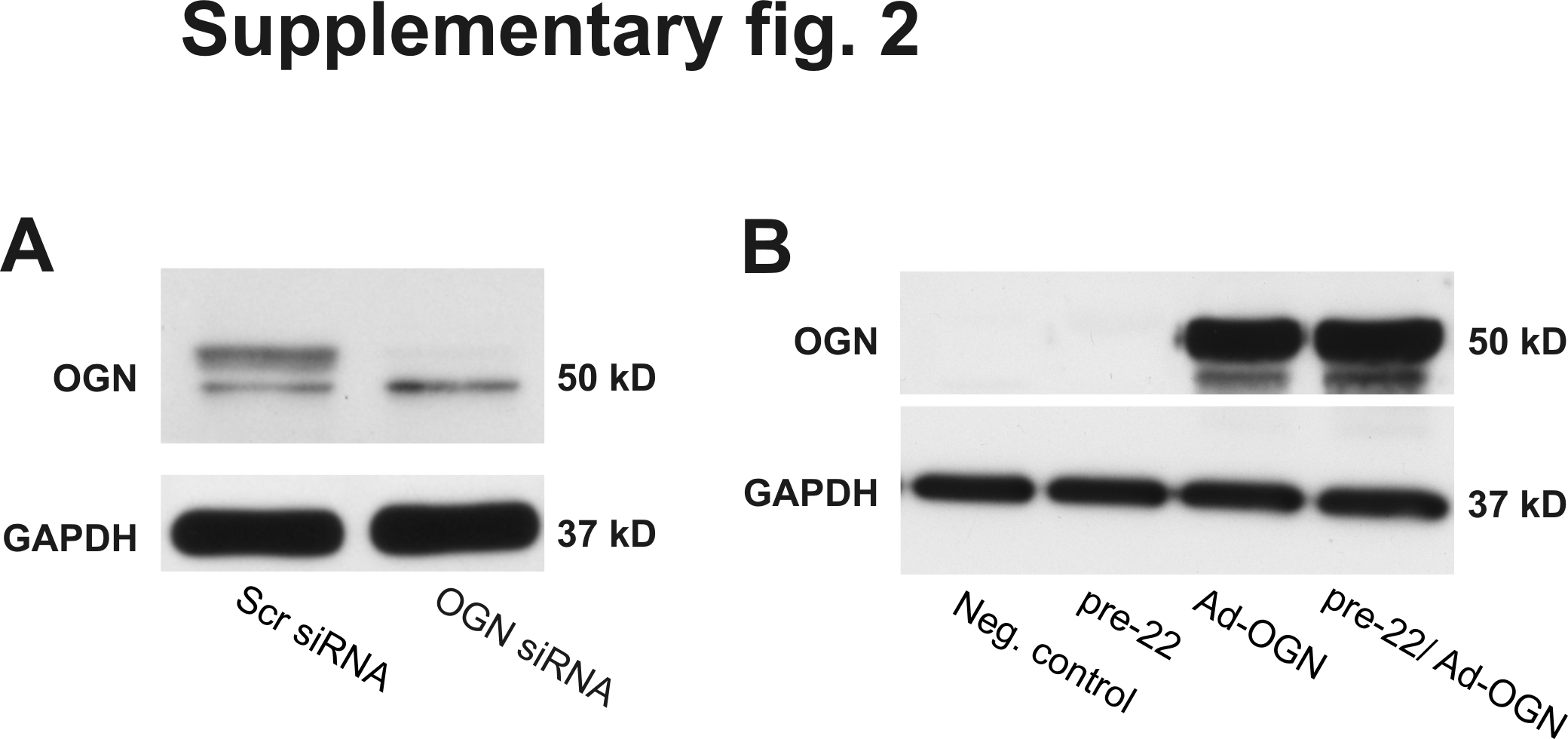


Supplementary Figure 3. SA-β-Gal staining after combinations of miR-22 and mimecan modulation in **cardiac fibroblasts.** Cells were co- transfected with control miR and Ad-GFP (negative control), miR-22 antagonist (anti-miR22) and OGN siRNA alone or in combination (OGN siRNA/ anti-miR22) and, subsequently, subjected to histochemical visualization of senescence- associated β-Gal activity. Likewise, cells were transfected with miR-22 and/or transduced with a mimecan construct (Ad-OGN) (B). Data are expressed as mean ± SEM (n= 4/ group); *, p< 0.05.


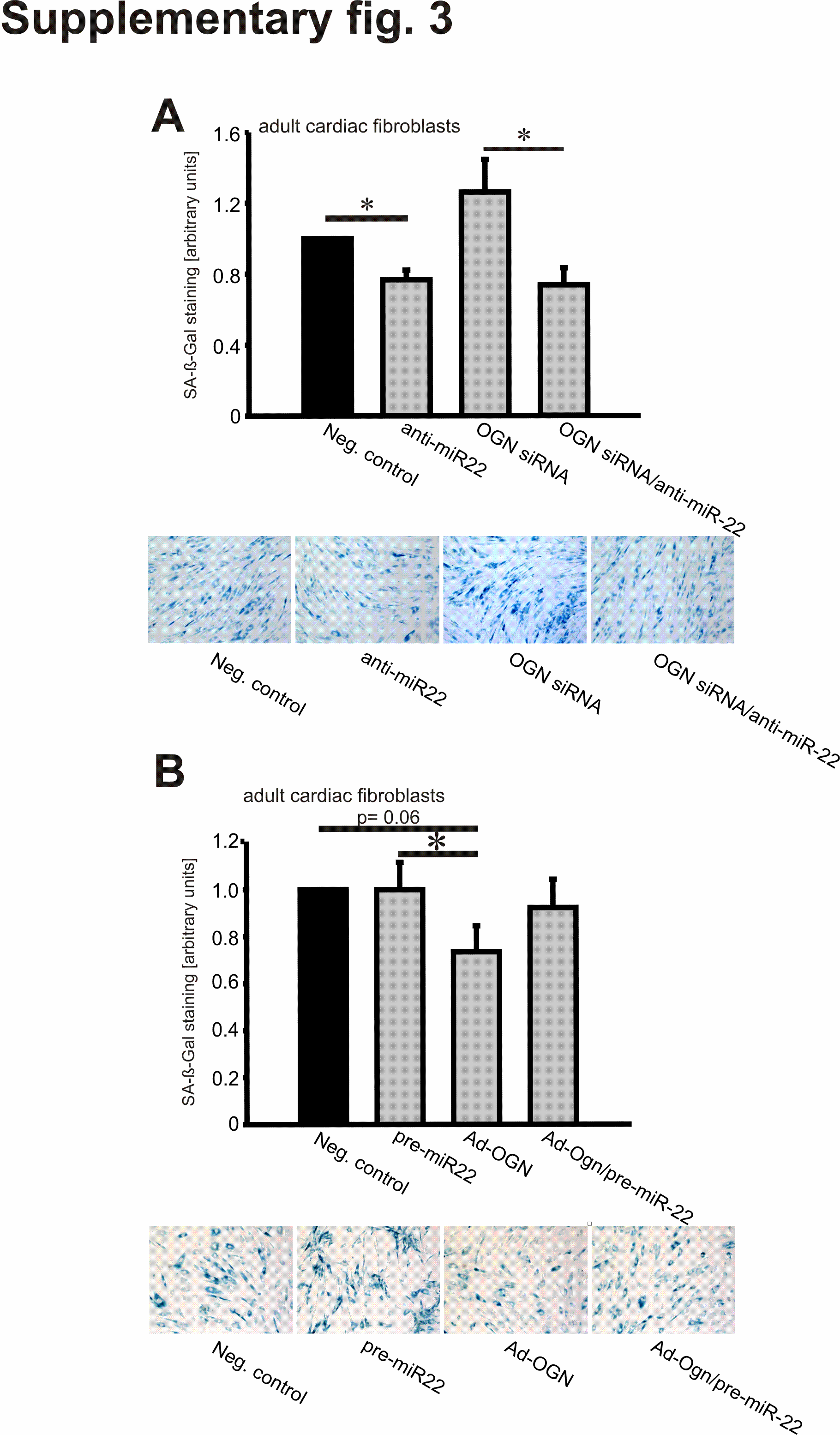


Supplementary Figure 4. Human cardiac fibroblast chemotactic migration after combinations of miR-22 and mimecan modulation. Cells were co- transfected with control miR and Ad-GFP (negative control), miR-22 antagonist (anti-miR22) and OGN siRNA alone or in combination (OGN siRNA/ anti-miR22) and, subsequently, subjected to the migration assay. Likewise, cells were transfected with miR-22 and/or transduced with a mimecan construct (Ad-OGN) (B). Data are expressed as mean ± SEM (n= 4/ group). **, p< 0.01.


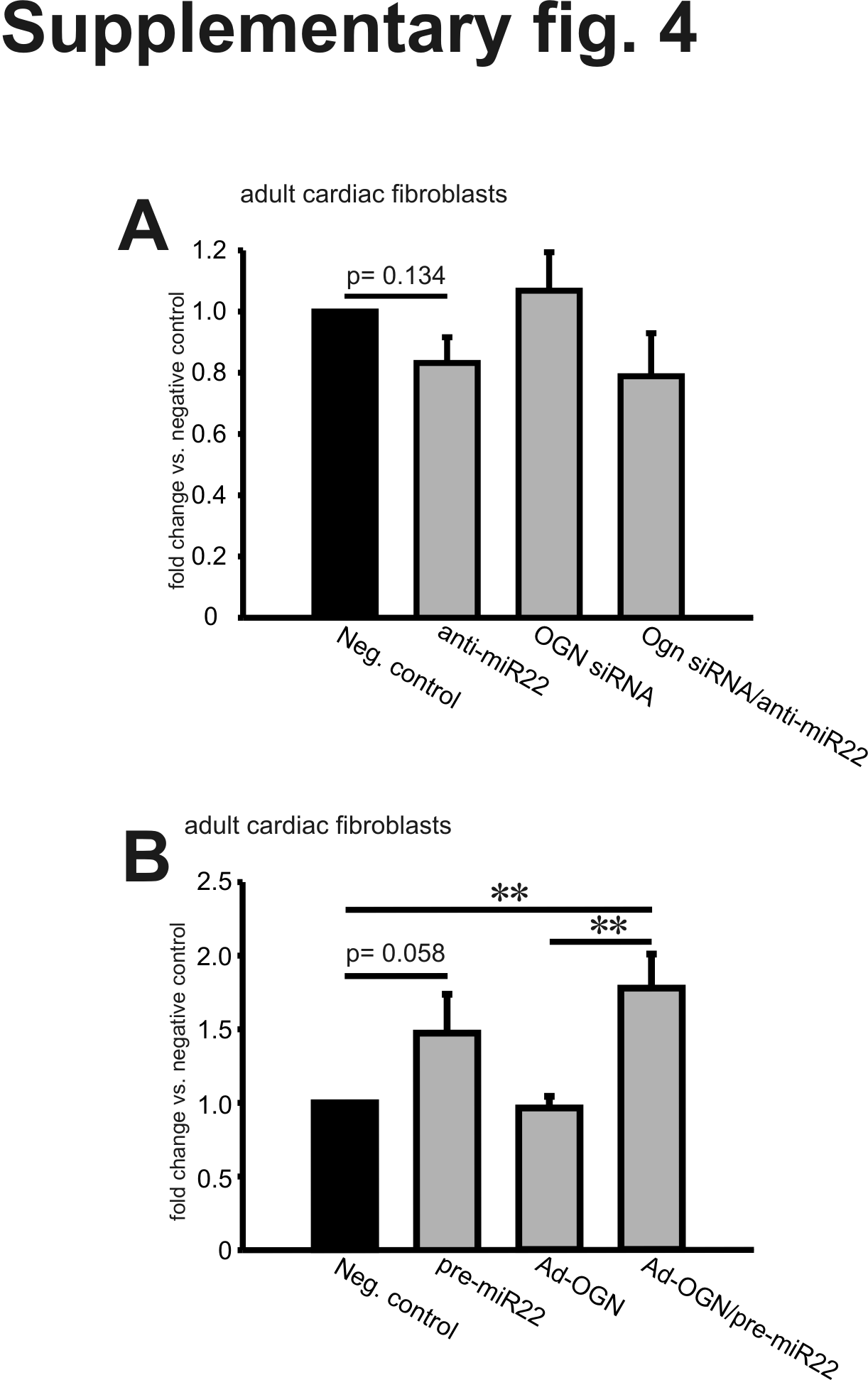


Supplementary Figure 5. Cellular senescence and chemotactic migration of smooth muscle cells. Human smooth muscle cells were transfected with control miR, miR-22 precursor or miR-22 antagonist. After 72h, the cells were incubated with β- Gal substrate to assess enzymatic activity of senescence- associated beta galactosidase (SA-β- Gal) (A) or subjected to migration through semi-permeable membrane towards 20% FCS (B). Data are expressed as mean ± SEM (n= 4-5/ group).


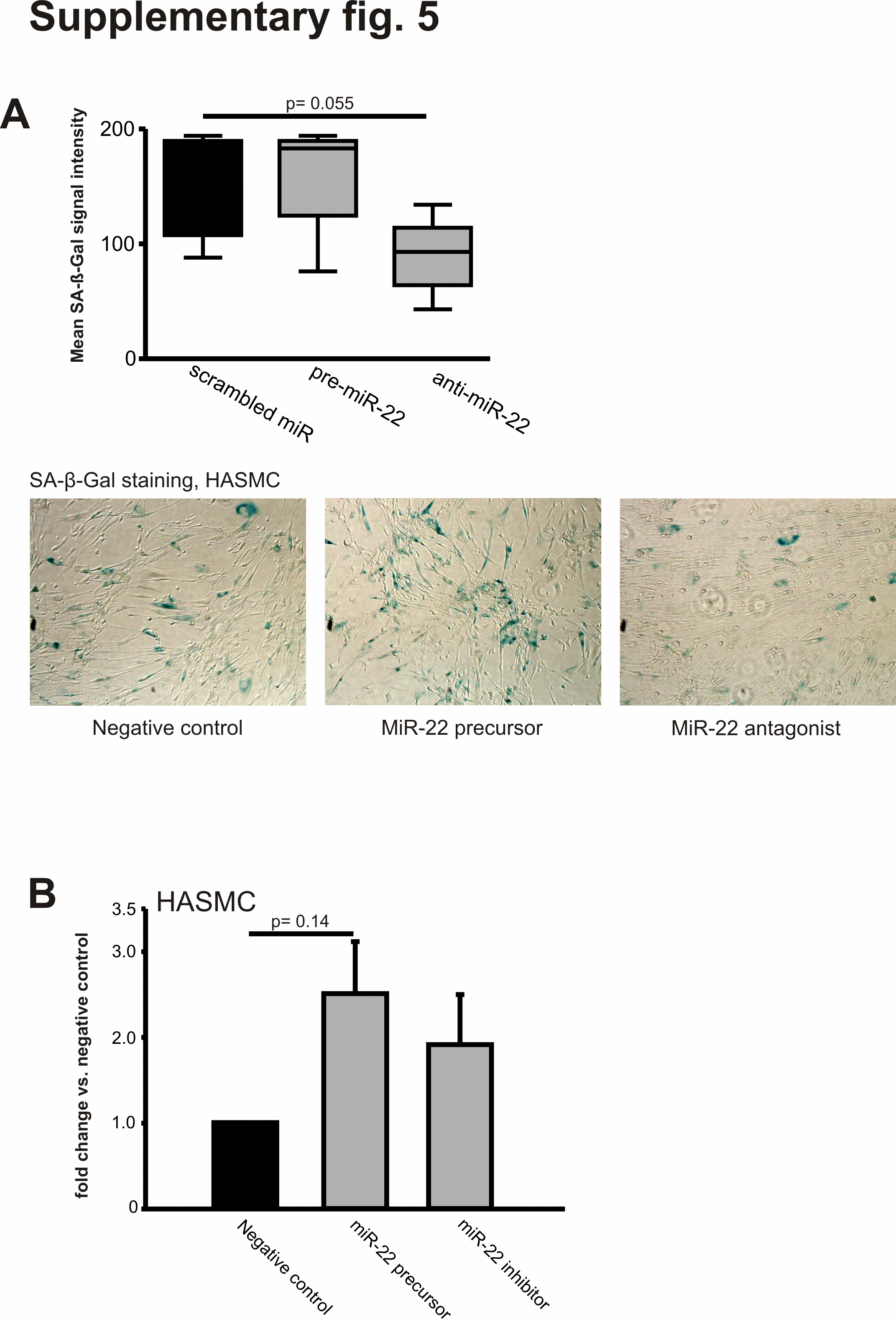


# Supplementary information

Materials and chemicals. HCF and HASMC, FCS and Fibroblast Basal medium 2 together with supplements (hFGF4 and insulin) and fibronectin were purchased by Promocell (Heidelberg, Germany). SmbM2 medium was purchased from Lonza (Cologne, Germany) and DMEM was obtained from PAA (Pashing, Austria). Mitomycin C was purchased from Applichem (Darmstadt, Germany). Trizol® reagent, Lipofectamine 2000 reagent and secondary antibodies donkey anti- goat- AlexaFluor488, donkey- anti- rat- AlexaFluor594, donkey- anti- rabbit AlexaFluor594 were purchased from Invitrogen (Darmstadt, Germany). Agilent RNA 6000 Nano Kit and QuikChange Multi Site-Directed Mutagenesis Kit were obtained from Agilent Technologies (Santa Clara, USA); “mirVana™ miRNA probe set” (#1564V1)and “mirVANA™ miRNA labeling kit” (#1562), control microRNA preNeg2, pre-miR-22 and anti-miR22 as well as pMIR-REPORT vector were purchased from Ambion (Austin, USA).The iScript™ Select cDNA synthesis system, iQ™ Supermix were obtained from Bio-Rad (Hercules, USA). Goat-anti-mimecan antibody was supplied by R&D Systems (Minneapolis, USA). Mouse anti-GAPDH, rabbit anti- smooth muscle actin and rabbit anti- SIRT-1 were obtained from Abcam (Cambridge, UK). Rat- anti- fibroblast marker, goat anti-p53 and mouse anti-p16 were obtained from Santa Cruz (Santa Cruz, USA) and rat- anti- CD31 was purchased from AbD Serotec (Raleigh, USA).. HRP- conjugated secondary antibodies anti- goat and anti- mouse together with Senescence β-Galactosidase Staining kit were purchased from Cell Signaling (Danvers, USA). Donkey serum was provided by Sigma- Aldrich (Munich, Germany). VectaShield® mounting medium was obtained from Vector Laboratories (Burlingame, USA). SiRNA against human osteoglycin and control siRNA (Trilencer) was provided by Origene (Rockville, USA). Adenovirus containing human OGN cDNA was obtained from SignaGen Laboratories (Rockville, USA). *SpeI* and *HindIII* restriction enzymes were purchased from New England Biolabs (Ipswich, USA). Beta-galactosidase control plasmid, luciferase and galactosidase substrates were purchased from Promega (San Luis Obispo, USA). BD Falcon Fluoro Block inserts with the 8 μm pore size were obtained from BD (Franklin Lakes, USA). WST-1 reagent and Annexin-V- FLUOS Staining system were purchased from Roche (Penzberg, Germany).
